# Prognostic impact of tumor size reduction assessed by magnetic resonance imaging after radiochemotherapy in patients with locally advanced cervical cancer

**DOI:** 10.3389/fonc.2022.1046087

**Published:** 2022-12-02

**Authors:** Abel Cordoba, Benedicte Durand, Alexandre Escande, Sophie Taieb, Mariem Ben Haj Amor, Marie Cecile Le Deley, Andree Michel, Florence Le Tinier, Delphine Hudry, Carlos Martinez, Eric Leblanc, Stephanie Becourt, Cyril Abdedaim, Lucie Bresson, Eric Lartigau, Xavier Mirabel, Fabrice Narducci

**Affiliations:** ^1^ Academic Radiotherapy Department, Oscar Lambret Center, Lille, France; ^2^ Radiology Department, Oscar Lambret Center, Lille, France; ^3^ Biostatistics Department, Oscar Lambret Center, Lille, France; ^4^ Medical Oncology Department, Oscar Lambret Center, Lille, France; ^5^ Surgical Oncology Department, Oscar Lambret Center, Lille, France; ^6^ Department of Surgical Oncology, Polyclinique Henin Beaumont, Henin, France

**Keywords:** locally advanced cervical cancer, tumor shrinkage, MRI, radiochemotherapy, brachytherapy

## Abstract

**Objective:**

Pelvic magnetic resonance imaging (MRI) is a key exam used for the initial assessment of loco-regional involvement of cervical cancer. In patients with locally advanced cervical cancer, MRI is used to evaluate the early response to radiochemotherapy before image-guided brachytherapy, the prognostic impact of which we aimed to study.

**Methods:**

Patients with locally advanced cervical cancer treated using concomitant radiochemotherapy followed by closure treatment between January 2010 and December 2015 were included in this study. Clinical, anatomopathological, radiological, therapeutic, and follow-up data were evaluated.

**Results:**

After applying the inclusion and exclusion criteria to the initially chosen 310 patients, 232 were included for evaluation (median follow-up period, 5.3 years). The median age was 50 years (range, 25–83 years), and the median tumor size was 47.5 mm (range, 0–105 mm). Based on the International Federation of Gynaecology and Obstetrics classification system, 9 patients were in stage IB2; 20, IB3; 2, IIA; 63, IIB; 4, IIIA; 7, IIIB; and 127, IIIC1 or higher. The re-evaluation MRI was performed at the median dose of 55.5 Gy, and median reduction in tumor size was 55.2% (range, −20–100%). There was a difference between the disease-free and overall survival rates of the patients with a tumor response greater or lesser than 50%. The risk of recurrence or death reduced by 39% in patients with a tumor size reduction >50%. The overall 5-year survival rate of patients with a response greater and lesser than 50% were 77.7% and 61.5%, respectively. The 5-year disease-free survival rate for these two groups of patients were 68.8% and 51.5%, respectively.

**Conclusion:**

Our study confirms the prognostic impact of tumor size reduction using MRI data obtained after radiochemotherapy in patients with locally advanced cervical cancer.

## Introduction

Cervical cancer is the fourth cause of cancer incidence and mortality worldwide (1). Survival rates largely depend on the cancer stage at diagnosis (2). Since 2018, the International Federation of Gynecology and Obstetrics (FIGO) staging system has been revised thanks to the evolution of imaging modalities and use of additional procedures in everyday practice (3, 4). Locally advanced cervical cancer comprises bulky tumors in FIGO stages IB3–IVA. The standard treatment for such cervical cancers is pelvic (and paraaortic, if indicated) radiochemotherapy that includes radiation therapy and concurrent cisplatin chemotherapy, followed by image-guided brachytherapy (5). Many factors, such as age, FIGO stage, tumor width, uterine corpus involvement, lymph nodes, and concurrent chemotherapy, are known to negatively impact survival outcomes (6–8). While therapeutic strategies, such as neoadjuvant and adjuvant chemotherapies, may prevent systemic recurrence (9, 10), local or loco-regional recurrences may be prevented with radical surgery (11).

Magnetic resonance imaging (MRI) is important during several stages of cervical cancer treatment. Standard MRI protocols include T1- and T2-weighted imaging of the pelvis in different planes. After revision of the FIGO classification system, MRI data are being considered while assessing the tumor stage. This imaging method is superior to clinical examination alone to assess the extent of tumor infiltration (12) and is particularly useful in determining the requirement of adaptative radiotherapy for cervical cancer (13). The extent of tumor shrinkage is considered to adjust the volume and dose in adaptive radiotherapy and helps define the volume for image-guided brachytherapy. Furthermore, the changes observed on MRI, such as those in apparent diffusion coefficient and signal intensity, can help predict outcomes after chemoradiotherapy for cervical cancer (14–16).

Therefore, an easy, reproducible test, such as MRI, is best suited in daily clinical practice to obtain information that can help in early identification of patients at high risk of local and loco-regional recurrence so that adjuvant therapies may be administered. We performed this study to test our hypothesis that tumor response assessed by MRI after concomitant radiochemotherapy for locally advanced cervical cancer has a prognostic impact on recurrence.

## Methods

### Patients and treatment

In this single-center, observational study, we included consecutive patients with a histologically proven diagnosis of locally advanced cervical cancer (FIGO stages IB2– IVA) who were treated with radiochemotherapy at our institution from January 2010 to December 2015. The inclusion criteria were as follows: age ≥18 years, cervix carcinoma observed on biopsy, and availability of MRI data before and after radiochemotherapy on the institution’s radiological picture archiving and communicating system at the time of the study. Patients who did not provide consent for the use of personal data according to the French national law regarding medical ethics in retrospective studies (Act no. 2012-300 of March 5, 2012) were excluded from the study. The treatment protocol that we implemented from 2010 to 2015 has been published previously (17, 18).

### Data collection

We retrospectively evaluated the initial demographic and clinical data, tumor characteristics, therapeutic data (radiation therapy and chemotherapy protocol), closure treatments (image-guided brachytherapy with or without radical surgery), and follow-up data that we obtained from medical records.

Radiological data, including maximum tumor diameter at the time of diagnosis, before image-guided brachytherapy, and after concomitant radiochemotherapy, were collected by performing MRI. MRI 1.5 T with gadolinium were performed at diagnosis and during the first week after completion of 45 Gy external beam radiotherapy prior to brachytherapy. Diffusion weighted imaging, and T1 and T2 weighted sequences with axial and sagittal planes acquired obliquely axed on the cervical canal were performed. Tumor size was defined as the largest tumor dimension measured on MRI T2 weighted sequences. All MRI examinations were evaluated by our radiologist specialized in female pelvic radiology.

Historically, in the gynecologic tumor committee of our center, MRI was performed the last week of radiochemotherapy in order to adapt the following treatment according to the response: Thus, for patients that tumor volume reduction was 50% or more, (estimated as maximum tumor diameter), treatment continued with uterovaginal brachytherapy and such patients were considered as good responders; on the other hand, those patients whose tumors had reduced in size by less than 50% were considered to be radioresistant and were offered surgical treatment 6-8 weeks after the end of radiochemotherapy.

Based in this argument, tumor size reduction rate of ≥50% was considered a satisfactory response and no tumor visualization was identified as a complete response.

### Outcomes

The primary endpoint was disease-free survival that was defined as the time period between the first day of radiochemotherapy and any recurrence of the tumor (local, regional, loco-regional, or metastatic) or death due to any cause. Data were censored when there was no recurrence or death during the last time the patient data were evaluated. The secondary endpoint was overall survival defined as the time period between the first day of radiochemotherapy and death due to any cause. Data were censored when the patients were alive during the last time the patient data were evaluated. Times until local, loco-regional, and metastatic recurrence were calculated. Toxicities were assessed using the Common Terminology Criteria for Adverse Events, version 4.

### Statistical analysis

Initial demographic and clinical data, treatment methods, survival and recurrence rates, and post-therapeutic complications were summarized using descriptive statistics. Missing data were specified. Initial categorical variables were expressed as numbers and percentages, while continuous variables were expressed as median (range) or mean (standard deviation). Disease-free and overall survival rates were assessed using the Kaplan–Meier method. The cumulative impact of local, regional or loco-regional, and metastatic recurrences was evaluated by the competing risks method described by Kalbfleisch and Prentice. To compare continuous and categorical variables, we used the Student’s t-test or Mann–Whitney U test and Fisher’s exact or chi-square test, respectively. The Fine and Gray model was used to compare the MRI data at the end of radiochemotherapy between responsive and non-responsive patients. The Cox regression analysis was used to test the association between various prognostic factors and disease-free survival. A p value <0.05 was considered statistically significant. All statistical analyses were performed using Stata Statistical Software, version 15 (StataCorp LP, College Station, TX, USA).

## Results

**Figure 1:**
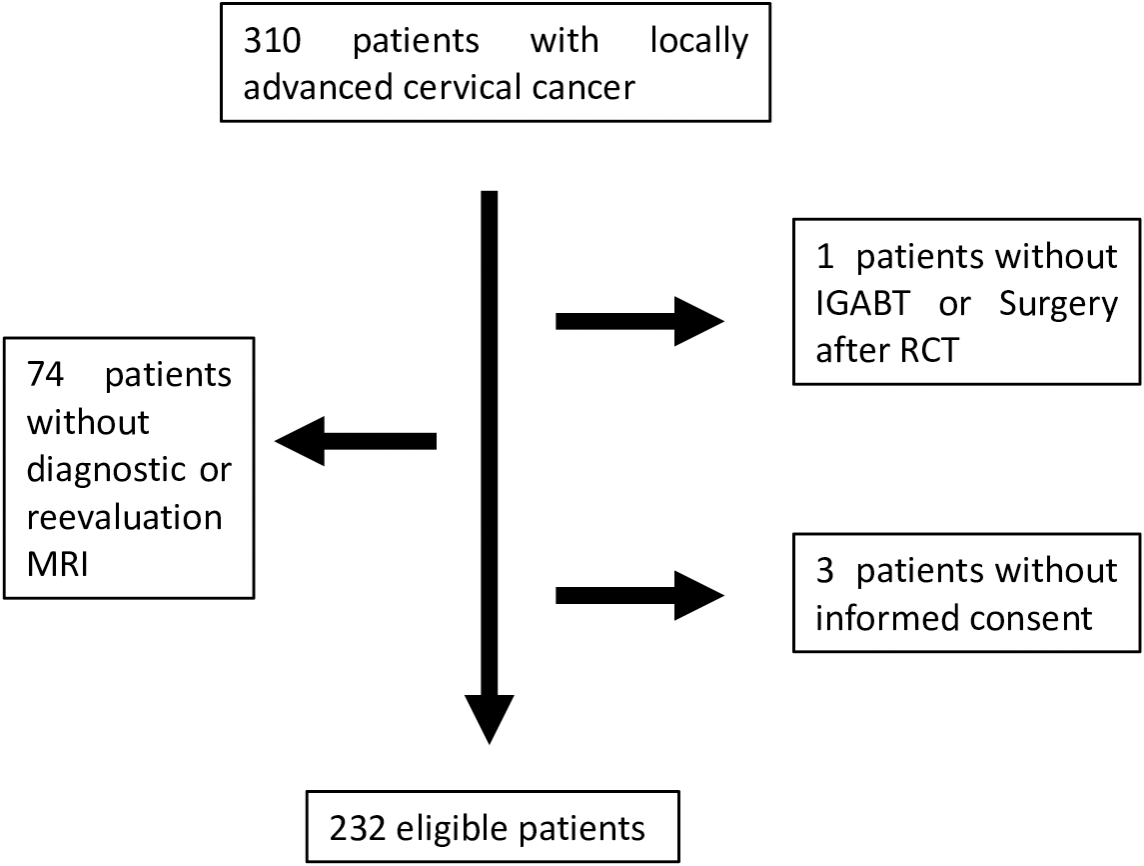
Flowchart showing patient selection.

The flowchart showing the selection criteria based on which 232 patients were included for evaluation is shown in **Figure 1**. The median follow-up period was 5.3 years.

### Clinical and tumor characteristics

At diagnosis, the median age was 50 years (range, 27–83 years) with a performance status of 0 for 193 patients (82.5%) and ≥1 for 41 (17.5%). Active smoking was reported by 83 patients (35%), 81 (34.9%) never smoked, 20 (8.6%) stopped before diagnosis, and data were not reported for 48 (20.7%). The main clinical symptom was metrorrhagia that was reported by 180 patients (77.5%), 22 (9.4%) had other symptoms, and 30 (12.8%) displayed no symptoms.

The FIGO classification stage was IIIC1 or higher for 127 patients (54.7%), IB2 for 9 (3.8%), IB3 for 20 (8.6%), IIA for 2 (0.7%), IIB for 63 (26.9%), IIIA for 4 (1.7%), and IIIB for 7 (2.9%). Histological examination revealed tumor type as squamous carcinoma for 192 patients (82.8%), adenocarcinoma for 34 (14.5%), and other types for 6 (2.5%). The median tumor size at diagnosis was 47.5 mm (range, 0–105 mm) (**Table 1**).

**Table 1:** Histological characteristics at diagnosis.

	Characteristics	N	%
FIGO stage			
	IB1	1	0.00%
	IB2	7	2.9%
	IB3	21	8.9%
	IIA	2	0.00%
	IIB	63	26.9%
	IIIA	4	1.7%
	IIIB	7	2.9%
	IIIC	59	25.2%
	IVA	42	17.9%
	IVB	20	8.5%
**Clinical size of tumor at diagnosis (mm)**			
	median (min, max)	40	(0, 100)
**Histological type**			
	Adenocarcinome	34	14.7%
	Epidermoid	192	82.8%
	Others	6	2.6%
**Grade differentiation**			
	1	43	18.5%
	2	54	23.3%
	3	42	18.1%
	Missing	93	40.1%

FIGO, Federation of Gynecology and Obstetrics.

### Response and outcomes

All the patients received concomitant chemotherapy; 210 (90.5%) received cisplatin weekly; 21 (9.0%), carboplatin; and 1 (0.4%), cisplatin–5-fluorouracil. The median overall treatment time was 50 days (range, 37–225 days).

**Figure 2:**
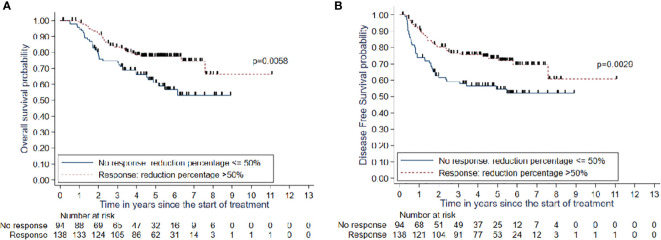
**(A)** OS and **(B)** DFS in patients with good response (>50%) or inadequate response (<=50%).

MRI was performed after radiochemotherapy (median radiation dose, 55.6 Gy). The median maximum tumor diameter before treatment was 47cm (0-105cm) and 21cm (0–53) after radiochemotherapy. The median tumor size reduction rate was 55.2% (−20–100%). Satisfactory response was observed in 139 patients (59.4%) and 29 (12.4%) demonstrated a complete response (**Figure 2**).

The risk of local recurrence after 1 and 5 years of follow-up since the beginning of treatment was 10% (95% confidence interval [CI]: 6.8%–14.7%) and 17% (95% CI: 12.5%–22.6%), respectively. In terms of recurrence-free survival, there were 78 events, namely, 67 recurrences followed by death, 11 recurrences without death, and 0 deaths without recurrences. The median time to recurrence-free survival was not achieved in this study after 11 years of follow-up since the start of treatment. The recurrence-free survival rates after 1, 3, and 5 years of follow-up since the start of treatment were 84.3% (95% CI: 78.9%–88.4%), 69.7% (95% CI: 63.2%–75.3%), and 66% (95%CI: 59.2%–72.0%), respectively. The overall survival rates after 1, 3, and 5 years of follow-up were 97.4% (95% CI: 94.3%–98.8%), 80.0% (95% CI: 73.9%–84.6%), and 70.7% (95% CI: 64.0%–76.5%), respectively.

Complete response was never obtained for 28 patients (12.1%) and 210 patients (89.7%) became disease free at one point. Recurrence was observed in 78 patients (33.3%) at a median time of 12.5 months (range, 3.2–69.5 months), i.e., 25 (32.1%) with local recurrences; 17 (21.8%), regional; 14 (17.9%), loco-regional; and 54 (69.2%), metastatic. While 67 patients (28.6%) died, 167 (71.4%) were alive at the end of the study.

There was a statistically significant difference in the 1- and 5-year overall survival rates between patients with a good tumor response and those with an inadequate tumor response (98.5% [95% CI: 94.2%–99.6%] and 77.5% [95% CI: 69.2%–83.8%] vs. 95.7% [95% CI: 88.8%–98.4%] and 61.0% [95% CI: 49.4%–70.7%]; p=0.005).

There was a statistically significant difference in the 1- and 5-year recurrence-free survival rates between patients with a good tumor response and those with a poor tumor response (91.1% [95% CI: 85%–94.9%) and 73.3% [95% CI: 64.6%–80. 2%] vs. (73.9% [95% CI: 63.7%– 81.7%) and 54.5% (95% CI: 43.2%–64.5%]; p=0.0029).

There was a significant rise in the 1- and 5-year risks of local recurrence in patients with an inadequate tumor response (18.5% [95% CI: 11.9%–28.0%] and 26.2% [95% CI: 18.1%–36. 9%]) than those of patients with a good tumor response (4.4% [95% CI: 2.0%– 9.5%] and 10.7% (95% CI: 6.5%–17.6%]; p=0.007).

### Prognostic factors

The characteristics that demonstrated a statistically significant association with disease-free survival in univariate analysis are shown in **Table 2**.

**Table 2:** Prognostic value for disease-free survival using univariate and multivariate analyses.

**Characteristics**	**N**	**Raw HR**	**95% CI**	**Raw p value**	**Adjusted HR**	**95% CI**	**Adjusted p value**
**Tumor response**	**234**						
>50%	**139**	0.52	0.3– 0.81	0.004	0.54	0.34– 0.85	0.008*
<50%	**95**	1			1		
**Age at diagnosis**	**234**	1.08	0.98– 1.17	0.087	0.97	0.88– 1.07	0.549
**OMS**	**234**						
>1	41	2.94	1.83– 4.72	<0.001	3.49	2.01– 6.03	<0.001*
0	193	1			1		
**FIGO stage**	**234**			0.1274			0.067
IB1–IIA	31	0.52	0.24– 1.16		0.55	0.33– 0.93	
IIB–IIIB	75	0.69	0.42– 1.14		0.64	0.29– 1.43	
IIIC1 and higher	128	1			1		
**Histological type**	**228**			0.008			0.005*
Adenocarcinoma	34	2.04	1.20– 3.47		2.16	1.25– 3.72	
Squamous carcinoma	194	1			1		
**Tobacco consumption**	**186**			0.2355			
Yes	82	0.7	0.43– 1.14				
Ceased	20	0.59	0.25– 1.40				
No	84	1					
**Clinical size of tumor at diagnosis**	126	1.01	0.99– 1.03	0.292			

HR, hazard ratio; 95% CI, 95% confidence interval; OS: FIGO, Federation of Gynecology and Obstetrics.

^*^p <0.05 in multivariate analysis.

Histologically, there was a statistically significant difference between patients with squamous cell carcinoma and those with adenocarcinoma both in in response (p=0.038) and overall survival rates (p=0.02). The median overall survival rate was observed after 6 years of follow-up since the start of treatment in the adenocarcinoma group, whereas patients with squamous cell carcinoma did not achieve median survival even after 10 years of follow-up. The overall survival after 5 years of follow-up in patients with squamous cell carcinoma was 72.1% (95% CI: 64.5%–78.3%) compared with 60.9% (95% CI: 42.2%–75.1%) in patients with adenocarcinoma.

Multivariate analysis showed that the tumor response rate after chemoradiotherapy had a statistically significant association with disease-free survival after adjustment (p=0.008). Recurrence or death risk showed a statistically significant reduction by 46% in patients with a satisfactory response than the patients without a satisfactory response did (hazard ratio [HR]=0.54, 95% CI: 0.34–0.85).

**Figure 3:**
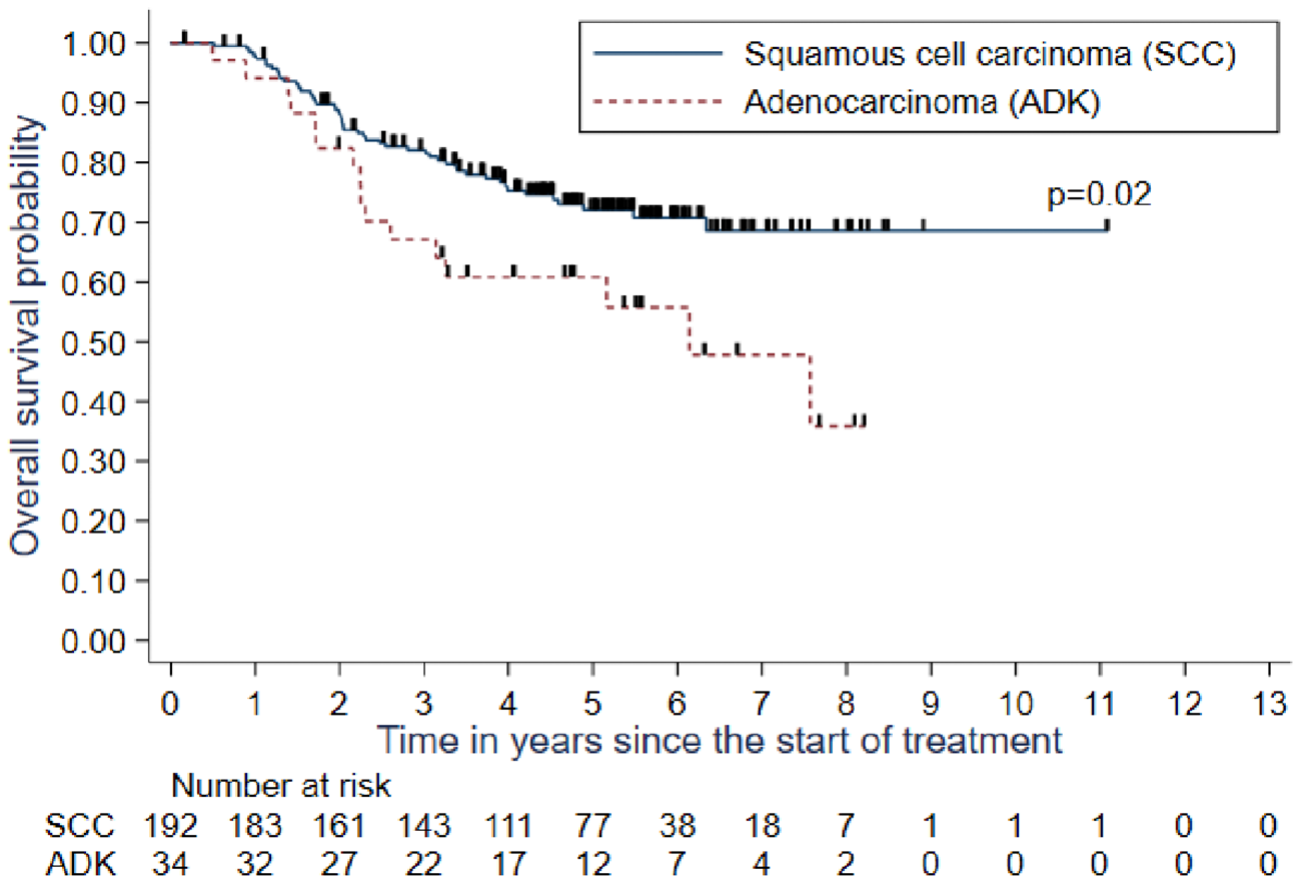
Overall survival (OS) depending histologic type.

The other prognostic factors associated with disease-free survival observed by multivariate analysis were performance status ≥1 (p<0.001), histologic type (p=0.005) and total time of treatment (p=0.003). Recurrence or death risk was three times higher in patients with a performance score ≥1 than in patients with a performance score of 0 (HR=3.49 [95% CI: 2.01–6.03], p<0.001). Furthermore, this risk was twice as high in patients with adenocarcinoma compared with that in patients with squamous cell carcinoma (HR=2.16 [95% CI: 1.25–3.72], p=0.005) (**Figure 3**). TTT superior to 50 days was associated in multivariate analysis to higher risk of relapse free survival (HR=2.26 [95% CI: 1.33–3.83], p=0.003)

## Discussion

### Summary of main results

In this study, we analyzed the MRI data of consecutive patients with locally advanced cervical cancer treated between 2010 and 2015. We found that the 1- and 5-years local control rates dropped significantly in patients with insufficient tumor response than those in patients with good. Furthermore, there was statistically significant reduction by 46% in recurrence or death risk in the patients with satisfactory response compared with that in those without a satisfactory response.

### Results in the context of published literature

MRI is the method of choice for local tumor staging at diagnosis and tumor response evaluation in cases of cervical cancer (19). Moreover, tumor size estimated by clinical palpation or using MRI measurements is an important prognostic factor in cervical cancer (20, 21). Recently, a European multi-institutional study has demonstrated that MRI-based image-guided brachytherapy is effective in providing local control and improving outcomes in patients with locally advanced cervical cancer (22). For selected elderly patients who cannot undergo uterovaginal brachytherapy, volumetric-modulated arc therapy with simultaneous integrated boost can be useful (23). Moreover, the dose to high-risk clinical target volume parameter and duration of treatment inferior to 50 days are correlated to local control (7).

Mazeron et al. found that a high-risk clinical target volume >30 cm^3^ was an independent factor for local control with a relative local relapse risk ratio of 2.51 (p=0.048), and in such cases, a dose of 92 Gy was required to achieve a 90% probability of local control. However, a dose of 73.9 Gy was administered in cases of volumes <30 cm^3^ (p =0.03) to achieve 90% local control (6). In a study by Potter et al., the 5-year local control rate was 92%; 5-year pelvic control rate, 87%; 5-year nodal control rate, 87%; 5-year overall survival rate, 74%; and 5-year disease-free survival rate, 68% (7). Dimopoulos et al. demonstrated a correlation between local control and dose to high-risk clinical target volume parameter depending on the residual tumor size after radiochemotherapy. The D90 for high-risk clinical target volume >87 Gy resulted in an LR incidence of 4% compared with 20% associated with D90 for high-risk clinical target volume <87 Gy (22).

Furthermore, MRI data have an important role in the revised cervical cancer FIGO classification system for local-regional tumor staging, evaluation of the response to treatment, and detection of tumor recurrence and possible complications (24) The recommendations of the Groupe Européen de Curiethérapie–European Society for Therapeutic Radiation and Oncology (GEC-ESTRO) group state that MRI is the main tool to identify the organs at risk and determine the target volume during uterovaginal image-guided brachytherapy in patients with locally advanced cervical cancer (25).

Similar to our findings, Schernberg et al. found that 247 patients with locally advanced cervical cancer treated with combined radiochemotherapy and image-guided brachytherapy demonstrated a reduction in gross tumor volume of at least 90%, which is correlated with reduced overall survival, progression-free survival, local control, and distant metastasis control (p<0.001). Reduction in gross tumor volume produces a survival impact that is greater than that by high-risk clinical target volume (26). EMBRACE group has also evaluated Local tumor regression evaluated by a T score, measured by MRI and clinical examination and they showed its local control predictive factor and also they demonstrated that it is useful to plan IGABT (27). This aspect is of utmost importance. Having the right information in terms of imaging with an MRI just before brachytherapy time will optimize the implant guided by a thorough clinical examination.

Angeles et al. evaluated the impact of tumor volume and regression after external beam radiotherapy measured by MRI on overall survival and relapse-free survival and found that tumor reduction rate ≥60% was significantly associated with a decreased risk of relapse and death (28). Nam et al. determined the impact of tumor regression measured by MRI at the beginning of radiotherapy or radiochemotherapy, mid-radiotherapy, and 1 month after completion of radiotherapy and found that patients with mid-radiotherapy regression ≥75% had 100% 5-year local control rates and better disease-free survival than those with mid-radiotherapy regression <75% (29). An important point to consider is whether tumor reduction should be assessed by regarding just the dimensions of the tumor or include volume measurements as well. In this study, we did not evaluate the tumor before treatment by diffusion-weighted MRI, which is known to be associated with local control and relapse-free survival of patients with locally advanced cervical cancer (14, 15, 30).

Concerning histologic type, adenocarcinomas have been historically described as radioresistant tumors compared with epidermoid carcinomas and are associated with poor clinical outcomes mostly due to incomplete tumor regression after radiochemotherapy (31). Our results showed an overall survival rate of 72.1% after 5 years of follow-up in patients with squamous cell carcinoma compared with 60.9% in patients with adenocarcinoma. However, a recent study based on the Surveillance, Epidemiology, and End Results database showed no difference in overall survival between patients with locally advanced cervical cancer having squamous carcinomas and adenocarcinomas (32).

### Implications for practice and future research

A question arises regarding the action required in cases of non-responding patients and those with high-volume tumors observed while performing image-guided brachytherapy. The policy at our center before 2015 was to debate whether surgery was an option. In this study, among the 15 patients who underwent surgery after radiochemotherapy, tumors were not observed in 7 patients. Moreover, surgery is associated with a high risk of urinary disorders and bowel problems. Thus, the current policy at our institution is to treat all such patients, including those responding late to treatment, with radiochemotherapy and three-dimensional image-guided brachytherapy independent of the percentage of tumor regression observed on MRI.

Furthermore, concomitant/adjuvant strategies based on the addition of immunotherapy agents, such as atezolizumab (NCT03612791) or cisplatin agents (NCT04016142), are being evaluated in prospective studies. Overall, our study proves that MRI is a useful tool in the management of patients with locally advanced cervical cancer and the mandatory performance of MRI after radiochemotherapy before image-guided brachytherapy should be considered.

### Strengths and weaknesses

The main strength of this study lies in the large, homogeneous study population and the long follow-up duration. Nevertheless, this study has a limitation, namely, the retrospective collection of data and emergence of late comorbidities. The current radiotherapy protocol has, therefore, been changed.

## Conclusions

Our study highlights the importance of tumor shrinkage after radiochemotherapy measured by MRI in determining prognosis, which conforms to the findings of previous studies.

## Data availability statement

The original contributions presented in the study are included in the article/supplementary material. Further inquiries can be directed to the corresponding author.

## Author contributions

AC, BD, FT, EL, XM and AE contributed to conception and design of the study. BD and AC organized the database. MD and AM performed the statistical analysis. AC and BD wrote the first draft of the manuscript. AC, LB, EL, CM, DH and FN wrote sections of the manuscript. All authors contributed to manuscript revision, read, and approved the submitted version

## Acknowledgments

We thank all the patients for giving us their consent.

## Conflict of interest

The authors declare that the research was conducted in the absence of any commercial or financial relationships that could be construed as a potential conflict of interest.

## Publisher’s note

All claims expressed in this article are solely those of the authors and do not necessarily represent those of their affiliated organizations, or those of the publisher, the editors and the reviewers. Any product that may be evaluated in this article, or claim that may be made by its manufacturer, is not guaranteed or endorsed by the publisher.
